# Changes in Retinol-Binding Protein Concentrations and Thyroid Homeostasis with Nonoccupational Exposure to DDT

**DOI:** 10.1289/ehp.1002616

**Published:** 2010-12-14

**Authors:** Rhena Delport, Riana Bornman, Una E. MacIntyre, Nicholette M. Oosthuizen, Piet J. Becker, Natalie H. Aneck-Hahn, Christiaan de Jager

**Affiliations:** 1 Department of Chemical Pathology, School of Medicine, University of Pretoria, Pretoria, South Africa; 2 Department of Urology, School of Medicine, University of Pretoria, Pretoria, South Africa; 3 University of Limpopo, Institute for Human Nutrition, Medunsa, South Africa; 4 Biostatistics Unit, Medical Research Council of South Africa, Pretoria, South Africa; 5 School of Health Systems and Public Health, University of Pretoria, Pretoria, South Africa

**Keywords:** DDT, endocrine disruptors, retinol-binding protein, thyroid homeostasis

## Abstract

**Background:**

The insecticide dichlorodiphenyltrichloroethane (DDT) has been used for malaria vector control in the northern and eastern parts of the Vhembe District of Limpopo Province, South Africa, since 1945. Bioaccumulation of DDT raises concern because it reportedly affects thyroid function.

**Objective:**

Our objective was to investigate the association between DDT uptake (as reflected in plasma concentrations) and thyroid homeostasis while considering related factors.

**Methods:**

We compared dietary intake, serum retinol-binding protein (RBP), transthyretin (TTR) and albumin concentrations, and liver and thyroid function between cases with evidence of a body burden of DDT in the circulation (concentration of any DDT isomer ≥ 0.02 μg/g lipid; *n* = 278) and controls (concentration of all DDT isomers < 0.02 μg/g lipid; *n* = 40) in a cross-sectional study. Further analyses were performed to assess the relevance of changes in RBP status associated with DDT uptake.

**Results:**

RBP concentrations below the reference range were more prevalent in cases (54% vs. 10% in controls; χ^2^ = 27.4; *p* < 0.001), which could not be explained by nutrient intake. We observed significantly lower thyroid hormone concentrations among cases (*p* ≤ 0.01). We also observed a significant linear trend for serum concentrations of free thyroxine and free triiodothyronine (*p* < 0.001) and a significant quadratic trend for serum thyroid-stimulating hormone (*p* = 0.025) and TTR (*p* < 0.001) across the control group and case groups with normal and relatively low RBP concentrations. Relatively low RBP concentrations were associated with significantly higher DDT and 1,1-dichloro-2,2-bis(*p*-chlorophenyl) ethylene (DDE) isomer concentrations and with a higher DDE/DDT ratio (*p* ≤ 0.01), which signifies long-term exposure. Inadequate intake of vitamin A and zinc were observed in 84% and 58%, respectively, of the total study population.

**Conclusion:**

RBP concentrations appear to decrease in the presence of long-term DDT uptake, which may have deleterious effects on thyroid function and vitamin A nutritional status. This is of major concern in a population with poor vitamin A and zinc intake.

The pesticide dichlorodiphenyltrichloroethane (DDT) and its metabolites are chemically stable and lipophilic in nature and have a propensity to bioaccumulate (Colborn et al. 1993; U.N. Environment Programme 2004) mainly as the metabolite 1,1-dichloro-2,2-bis(*p*-chlorophenyl) ethylene (*p*,*p*′-DDE) (Smith 1991). Plasma *p*,*p*′-DDE concentrations thus reflect exposure from all sources over the previous years (Hauser et al. 2003), and high concentrations of *p*,*p*′-DDT and low DDE/DDT ratios are indicative of more recent exposure to DDT (Ahlborg et al. 1995). In rural parts of the Vihembe District, Limpopo Province, South Africa, DDT indoor residual spraying is used as a strategy to eradicate malaria, and endocrine-disrupting effects have been reported to be associated with the intervention (Aneck-Hahn et al. 2007; Bornman et al. 2010). However, effects of long-term DDT exposure on thyroxine (T_4_) transport have not yet been investigated in this population group. Because the retinol transport complex transports both retinol and T_4_, in this study we aimed to examine changes in retinol-binding protein (RBP), transthyretin (TTR), and thyroid hormone concentrations, while considering related dietary and other factors.

Assessment of thyroid function is of vital importance in this population, especially in vulnerable groups such as pregnant women and infants (Nagayama et al. 2007). Vitamin A status, however, also needs to be evaluated because vitamin A and its derivatives play an essential role in numerous biological functions, including vision, reproduction, growth and differentiation, and immunity. We measured RBP concentrations as a surrogate for serum retinol concentrations because RBP and retinol exist in approximately equimolar concentrations (Gamble et al. 2001). The main circulating form of vitamin A is all-*trans*-retinol, which occurs in plasma bound to RBP. RBP solubilizes and stabilizes the hydrophobic retinol and forms a 1:1 molar complex with TTR. Binding to TTR is thought to protect the small RBP molecule (21 kDa) from filtration by the renal glomeruli. RBP is produced mainly by the liver, but small amounts are synthesized in other tissues, including the eye. Extrahepatic synthesis may be important for recycling of retinol back to the liver or for uptake by tissues with a blood–tissue barrier (testis, eye, brain) (Quadro et al. 2003). It is becoming increasingly clear that RBP is not merely a carrier protein; it is also involved in regulating the transport, metabolism, and action of retinoids (Noy 2000). Studies in a knockout mouse model (RBP^−/−^) have shown that RBP is essential to mobilize retinol from hepatic stores and transport it to target tissues when dietary intake of vitamin A is inadequate. In the fasting state, 99% of vitamin A in plasma occurs as retinol–RBP. In the postprandial state, retinyl esters packaged into chylomicrons and their remnants, or bound to circulating lipoproteins, are an important RBP-independent source of vitamin A (Quadro et al. 2003). When dietary intake of vitamin A is low, acquisition via the postprandial route is minimal, making mobilization of retinol from hepatic stores by RBP the only means to supply tissues with the retinoids required for biological processes. Thus, our purpose in the present study was to investigate to what extent DDT uptake is associated with changes in thyroid and vitamin A homeostasis.

## Materials and Methods

The Ethics Committee of the Faculty of Health Sciences, University of Pretoria (reference 43/2003), and the Limpopo Provincial Government Department of Health and Social Development approved the research protocol (11 July 2002) for a study to assess the effect of DDT exposure on male fertility and general health parameters. This study was performed in compliance with the Declaration of Helsinki ethical principles for medical research involving human subjects. All volunteers provided written informed consent and were included in the study if they met the inclusion criteria as previously published by Aneck-Hahn et al. (2007). The subjects were 18- to 40-year-old nonoccupationally exposed males of Venda ethnicity who had lived in the communities for at least a year. Participants with a history of testicular trauma, orchitis, urinary infection, sexually transmitted diseases, neuropsychiatric disorders, use of hormonal medication, or exposure to known gonadotoxins were excluded from the study. The entire study population consisted of 546 male subjects, and samples were collected between November 2003 and October 2006 during six 5-day visits to the study area. The venous blood samples were collected between 0800 hours and 1000 hours, centrifuged at 670 × g for 10 min at room temperature, stored in 500-μL aliquots at −20°C on site, and then transferred to a 70°C freezer until analysis. Blood samples were collected as part of the ongoing study. RBP concentrations were determined in a subgroup of participants (*n* = 319) sequentially enrolled during four of the six visits. Cases (*n* = 279) were defined in this study as having evidence of DDT uptake (DDT isomer concentrations ≥ 0.02 μg/g serum lipid), whereas controls (*n* = 40) had no evidence of DDT uptake (DDT isomer concentrations < 0.02 μg/g serum lipid).

For the present study, we selected relevant data (e.g., duration of exposure, occurrence of common diseases and conditions) from the DDT exposure questionnaire used in the previously reported study (Aneck-Hahn et al. 2007). Body weight and height, measured using standardized techniques as part of the previous study, were used to calculate body mass index (BMI). Dietary intake data were collected using a semiquantitative food frequency questionnaire (SQFFQ), which was modified from a previous study (MacIntyre et al. 2002) and tested for validity against four 24-hr recalls in the target population. Food-intake data from the SQFFQ were analyzed for energy and nutrient content using FoodFinder 3 dietary analysis software of the South African Medical Research Council (Medical Research Council of South Africa 2010). Food intake was further analyzed into 15 food groups for analysis of food consumption patterns. Estimated average requirement (EAR) values (Food and Nutrition Board 2000) were used as cut points to define inadequate vitamin A and zinc intake. The respective EAR values for vitamin A and zinc intake were 625 retinol equivalents (REs) and 9.82 mg/day, respectively. Energy, nutrient, and food group intakes were available for 470 subjects in the total study population (*n* = 546). Dietary intake reports were obtained for all 40 controls and for 227 cases.

Serum samples were analyzed for RBP, TTR, liver enzymes, and albumin concentrations. Serum RBP was determined on an Immage nephelometer (Beckman-Coulter Ltd., Johannesburg, South Africa) using the Dade Behring RBP kit (Dade Behring, Marburg, Germany); the reference range was reported as 34–77 mg/L in serum. The limit of detection (LOD) of the method, as established in our laboratory, was 20 mg/L, and 45 cases had RBP concentrations < LOD. Subjects within the case group were categorized as those having either relatively low (RBP < 34 mg/L; *n* = 151) or normal (≥ 34 mg/L; *n* = 128) RBP concentrations. Serum TTR was determined with the Beckman-Coulter TTR (prealbumin) kit (Beckman-Coulter, Ltd., Carlsbad, CA, USA) on an Immage nephelometer, and the reference range was reported as 18–45 g/L. Serum albumin and liver enzymes were determined on an LX-20 automated analyzer (Beckman-Coulter Ltd.). We performed thyroid function tests using an Immulite 2000 Analyzer (Siemens Southern Africa, Halfway House, South Africa). For black males 18–40 years of age, our laboratory used the following reference ranges: serum-free T_4_ (fT_4_), 9.2–26.0 pmol/L; serum-free triiodothyronine (fT_3_), 2.2–5.4 pmol/L; and thyroid-stimulating hormone (TSH), 0.47–4.70 mIU/L.

We performed exposure assessment as described previously (Aneck-Hahn et al. 2007). Levels of DDT and its metabolites were determined by the Residue Laboratory of the Veterinary Institute, Agricultural Research Council (Pretoria, South Africa; Facility Accreditation no. V0002, South African National Accreditation System, in accordance with ISO/IEC 17025:2005 guidelines) using a Shimadzu GCMS-QP2010 gas chromatograph/mass spectrometer (Shimadzu, Tokyo, Japan). Concentrations of DDT compounds in the plasma were expressed on a lipid-adjusted basis (micrograms per gram). The LOD for *p*,*p*′-DDT and *p*,*p*′-DDE was 0.02 μg/g lipid. Total cholesterol and triglycerides were determined by enzymatic methods, and the total plasma lipid concentration was calculated according to the formula of Rylander et al. (2006a):


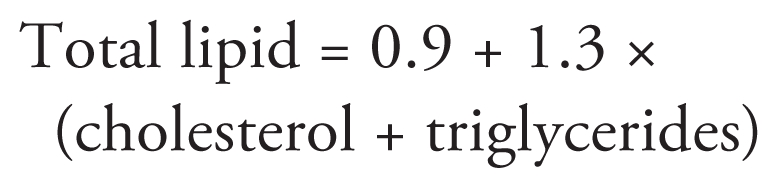


The DDT/DDE ratio was calculated as *p*,*p*′-DDT/*p*,*p*′-DDE.

We used Statistix 9 (Analytical Software, Tallahassee, FL, USA) to perform statistical analyses. Data were exposed to exploratory analysis to identify statistical outlier values and assess normality of the data. Appropriate transformations were made to the data where necessary. Student’s *t*-test was employed for between-group comparisons, taking account of the equality of variance, and when appropriate, nonparametric analyses were performed using the Wilcoxon rank-sum test. Analysis of variance was used on ranked data to compare unexposed subjects and subjects within the two exposed subgroups. Multiple comparisons employed Fisher’s least significant differences to make pairwise comparisons among the three groups (α = 0.5). Polynomial contrasts were used to assess the presence of trend. The chi-square test was used to test for association when data were categorical, and Pearson’s (product-moment) correlation coefficient was calculated to indicate the degree of linear association between continuous variables.

## Results

We excluded one exposed subject with hyperthyroidism (serum fT_4_, 16.2 pmol/L; serum fT_3_, 21.5 pmol/L; TSH, 0.05 mIU/L) from the study. The median (interquartile range) concentrations for the respective DDT isomers (micrograms per gram lipid) in cases were as follows: *o*,*p*′-DDT, 0.37 (0.00–1.25); *p*,*p*′-DDT, 5.66 (1.54–22.40); *o*,*p*′-DDD [1,1-dichloro-2-(2-chlorophenyl)-2-(4-chlorophenyl) ethane], 0.00 (0.00–0.28); *p*,*p*′-DDD, 0.00 (0.00–0.50); *o*,*p*′-DDE, 0.00 (0.00–0.17); and *p*,*p*′-DDE, 34.12 (0.71–120.78). The median (interquartile range) was 42.75 (3.11–141.34) μg/g lipid for the sum of all DDT isomer concentrations (∑DDT) and 2.77 (0.32–5.68) for the *p*,*p*′-DDE/*p*,*p*′-DDT ratio. Mean ages differed slightly but statistically significantly (Table 1). We regarded the difference in age as clinically insignificant for all analytes that we measured.

We observed relatively low RBP concentrations in 54% of cases and in 10% of controls (χ^2^ = 27.5; *p* < 0.001; Table 1). Serum albumin and TTR concentrations did not differ between the groups, whereas we observed significantly lower serum fT_4_, fT_3_, and TSH concentrations in cases. Both serum fT_4_ and TTR were significant cofactors for RBP, but statistical adjustment for these variables did not affect the significance.

Mean serum γ-glutamyltransferase (GGT) concentrations were significantly elevated with plasma DDT detection. Alcohol intake, as recorded in the SQFFQ, did not differ between groups. Furthermore, we observed no significant differences between exposure groups concerning BMI and intakes of total energy, protein, fat, and carbohydrate. The diet of the sample comprised primarily maize meal porridge [consumed by virtually the entire sample (99.6%) and in large amounts] and brown bread (consumed by 99%). Animal protein was consumed by subjects in the study sample (red meat, 64%; fish, 67%; chicken, 89%), in small amounts (median intake of combined flesh protein sources = 41 g/day). Only four subjects reported no intake of flesh sources whatsoever. Fruit and vegetables were consumed by > 90% of the sample, in relatively small amounts (median intakes of 100 and 50 g/day, respectively). Although a number of indigenous wild vegetables grow in the study area, these were consumed by only 60% of the study sample, in small amounts (median = 26 g/day). Fruit and vegetables contributed a mean ± SD of 57 ± 31% of the vitamin A intake. We found no statistically significant differences between groups with and without evidence of DDT uptake for any of the food intake variables (*p* > 0.05).

Table 2 lists trends in variables relating to thyroid hormone transport across the control group and case subgroups that had either normal or relatively low RBP concentrations using polynomial contrasts. For mean TTR concentrations, we observed a significant quadratic trend (*p* < 0.001) across the study groups but a significant linear trend (*p* < 0.001) for both serum fT_4_ and fT_3_ concentrations and a significant quadratic trend (*p* = 0.025) for serum TSH concentrations.

Comparison of DDT isomers within the group with DDT uptake between subjects with relatively low RBP and normal RBP concentrations showed that significantly higher (*p* ≤ 0.01) concentrations of the DDT and DDE isomers and ∑DDT were associated with subjects with relatively low RBP concentrations (Table 3) based on comparisons performed on log-transformed data. Furthermore, ratios of *p*,*p*′-DDE/*p*,*p*′-DDT, representing recent exposure to the parent compound *p*,*p*′-DDT, were significantly higher (*p* < 0.001) in subjects with low RBP concentrations. Within the exposed group, childhood exposure to DDT was associated with low RBP concentration (χ^2^ = 7.74; *p* = 0.005): Only 25% of subjects with normal RBP concentrations were exposed to DDT during childhood, whereas 50% of subjects with relatively low RBP concentrations reported childhood exposure. However, breast-feeding as an infant (without consideration of parity) was not associated with RBP status.

We observed inadequate vitamin A intakes in 85% of subjects with no evident DDT uptake and in 81% of subjects with evidence of DDT uptake (χ^2^ = 0.32; *p* = 0.571), whereas zinc intakes below the EAR of 9.82 mg/day were present in 62% and 69% of the subject groups, respectively (χ^2^ = 0.70; *p* = 0.402). We observed concurrent inadequate intakes of vitamin A and zinc in 56% of unexposed and 60% of exposed subjects (χ^2^ = 0.74; *p* = 0.690). Protein malnutrition, defined as serum albumin concentrations < 35 g/L, as proposed elsewhere (Dati et al. 1996), was found in approximately 2.5% of all subjects.

Present or past occurrence of any of the following conditions were either reported by subjects or observed during the physical examination (numbers of subjects with low RBP concentrations per total observed for each condition ares shown in parentheses): fever within the preceding 3 months (6 of 9), malaria infection (8 of 10), tuberculosis (1 of 2), HIV/AIDS (0 of 0), sexually transmitted diseases (5 of 14), urinary tract infection (16 of 23), orchitis (9 of 33), epididimitis (1 of 1), sinusitis (2 of 2), and allergies (7 of 11). None of these conditions was significantly associated with RBP status in the exposed group.

## Discussion

The major findings after evaluation of DDT uptake-associated changes in retinol and T_4_ transport are that RBP concentrations below the reference range were more prevalent and circulating thyroid hormones are lower with DDT uptake (Table 1). Relatively lower RBP concentrations were associated with significantly higher DDT and DDE isomer concentrations and a higher *p*,*p*′-DDE/*p*,*p*′-DDT ratio, the latter signifying long-term DDT exposure.

Nutritional factors did not confound the RBP finding, and correction for significant covariates did not change the differences between cases and controls. Furthermore, relatively low RBP concentrations were associated with significantly higher DDT and DDE concentrations (Table 3). The higher *p*,*p*′-DDE concentrations and DDE/DDT ratios observed in the subjects with low RBP status signify long-term exposure to DDT in the group with low RBP concentrations (Ahlborg et al. 1995). This finding was confirmed by reported childhood exposure to DDT that was significantly associated with RBP status. Low RBP concentrations do not appear to be of clinical relevance in this apparently healthy study population, because we observed no significant association between RBP status and disease occurrence, as reported by subjects or as diagnosed during the clinical examination.

The food intake of the study sample is typical of that described for other rural South African populations, with the staple food (maize meal porridge) providing the bulk of the energy and nutrient intake (MacIntyre et al. 2002; Vorster et al. 1994). Although protein intake appeared to be adequate, foods of animal origin including milk, red meat, chicken, fish, and eggs were consumed in small amounts, resulting in low intake of high biological-value protein. The finding that 2.5% of the sample was protein deficient could be a reflection of the poor intake of high-biological-value proteins. Because foods of animal origin, particularly red meats, are rich sources of preformed vitamin A and zinc, their limited consumption could have contributed to the inadequate intakes of these micronutrients. Although a variety of indigenous and cultivated vegetables and fruit are found in the study area—including those rich in β-carotene such as indigenous spinach and cultivated pawpaws and mangoes—intake was low, which contributed to the low vitamin A intake (84% of all subjects did not meet EAR-defined levels of intake for vitamin A). In general, the zinc intake was poor in the population: The average daily intake of zinc was below the EAR in 65% of the total study population. Concurrent vitamin A and zinc deficiency was evident in 58% of the study population. Zinc deficiency leads to a reduction in plasma retinol and increases in hepatic retinol, and if the zinc deficiency is accompanied by a vitamin A–deficient diet, a synergistic effect on retinol homeostasis is exerted (Boron et al. 1988; Solomons and Russell 1980), which is a major concern in this population.

Estimation of nutrient intake is very difficult, and the reported vitamin A and zinc intakes might not be accurate reflections of the true intakes of the study sample. A validation study showed that the SQFFQ overestimated vitamin A intakes compared with multiple 24-hr recalls (MacIntyre and Bornman 2004). Furthermore, because approximately 60% of the vitamin A intake was derived from β-carotene, the available vitamin A was likely to be lower than reflected by the intake data. Therefore, it is unlikely that higher vitamin A intakes than those reported could have accounted for the RBP levels not being as low as expected for a vitamin A–deficient diet. In an earlier study in the same region, Vorster et al. (1994) reported a similar finding of adequate RBP levels, despite possibly inadequate vitamin A intakes.

Serum retinol concentrations are homeostatically controlled and do not begin to decline until liver reserves of vitamin A are critically low, which is approximately < 20 μg/g liver (Tanumihardjo 2004). Secretion of RBP from the liver is regulated by availability of retinol. Vitamin A deficiency leads to accumulation of RBP in the endoplasmic reticulum of hepatic parenchymal cells and decreased circulating concentrations (Noy 2000). Despite evidence of a vitamin A–deficient diet in both cases and controls, only 10% of the control subjects had relatively low serum RBP concentrations, whereas 54% of cases had relatively low concentrations. This suggests that another factor besides dietary vitamin A deficiency must have contributed to reductions in circulating RBP. We postulate that this factor is DDT. A number of mechanisms whereby xenobiotics may interfere with the metabolism and action of steroid ligands and their binding proteins have been reported in the literature,including *a*) dissociation of the RBP–TTR complex with subsequent loss of RBP by glomerular filtration (Brouwer et al. 1998); *b*) induction of cytochrome P-450 and UDP-glucuronosyl transferase hepatic enzyme systems with increased catabolism and release of vitamin A from the liver (Rolland 2000); and *c*) interference (agonistic and antagonistic) with the binding of natural steroid ligands to their receptors, including androgen, estrogen, and retinoic X receptors (Danzo 1997; Li et al. 2008). As discussed above, in subjects with vitamin A–deficient diets, RBP has a major role to ensure that retinoids reach target tissues. Further reductions in RBP attributable to DDT would be expected to aggravate an already tenuous situation with respect to retinoid-dependent processes.

TTR is closely associated with the vitamin A–RBP complex (Gamble et al. 2001), and the RBP/TTR ratio can be used for the indirect assessment of vitamin A status; a decreased ratio reportedly signifies a vitamin A deficiency (Rosales and Ross 1998). In the present study, 46 subjects had undetectable RBP concentrations, so calculation of RBP/TTR ratio was not possible. However, we observed that low TTR concentrations accompanied low RBP concentrations (Table 2) in the exposed group, which may imply that TTR concentrations will only decrease if RBP concentrations are extremely low, possibly due to DDT uptake in the body. Low TTR concentrations were reported previously for patients with low hepatic vitamin A stores (Rosales et al. 2002). This may explain our observations because subjects with low RBP concentrations have higher DDT. We also observed high DDT isomer concentrations in the group with the poor RBP status; the estrogenic potential of these isomers may further contribute to the low TTR concentrations because *TTR* mRNA expression is down-regulated by estrogenic exposure (Urbatzka et al. 2007).

The changes in thyroid function that were associated with DDT uptake is of concern. Serum fT_4_, fT_3_, and TSH concentrations were significantly lower in the presence of DDT uptake (Table 1). This finding appeared to relate to RBP status as well as a significant trend observed for serum levels of fT_4_, fT_3_, and TSH concentrations (Table 2) across the study groups consisting of subjects with no evidence of DDT uptake and subjects with evidence of DDT uptake who had normal or relatively low plasma RBP concentrations. Inverse correlations between serum fT_4_ and fT_3_ and DDT concentrations have been reported previously, although findings across studies are not consistent (Meeker et al. 2007; Rylander et al. 2006b). Several mechanisms of thyroid disruption due to environmental chemicals have been proposed (Boas et al. 2009). The observed changes in circulating thyroid hormone concentrations may not be of clinical significance for relatively healthy male subjects because all concentrations are still well within the reference ranges for the age group and sex of our study participants, but vulnerable groups in the population (e.g., pregnant women and infants) may need closer monitoring.

## Conclusion

RBP concentrations appear to decrease in the presence of DDT uptake, and RBP status is associated with an increase in the plasma *p*,*p*′-DDE/*p*,*p*′-DDT ratio, which is indicative of long-term DDT exposure. The retinol transport complex appears to be affected, as is evident from changes in circulating thyroid hormone and TTR concentrations. This is of major concern in a population with poor vitamin A and zinc intake. Vector control with DDT, albeit effective, needs to be reconsidered in the light of these findings.

## Figures and Tables

**Table 1:** Nutritional and health-related variables between subjects with and without evidence of DDT uptake (mean ± SD).

	DDT isomer concentration	
Variable	≥ 0.02 μg/g lipid (*n* = 40)	< 0.02 μg/g lipid (*n* = 278)
Relatively low RBP concentration [*n* (%)]^a^#	4 ± 10	151 ± 54
Albumin (mg/L)	45.17 ± 4.19	44.98 ± 5.76
TTR (mg/L)	23.74 ± 5.74	23.51 ± 6.34
Serum fT_4_ (pmol/L)#	15.55 ± 2.10	13.00 ± 2.38
Serum fT_3_ (pmol/L)#	5.55 ± 0.78	4.84 ± 1.20
Serum TSH (mIU/L)*	2.67 ± 1.32	2.09 ± 1.09
Serum AST (IU/L)	31.17 ± 7.97	29.80 ± 9.48
Serum ALT (IU/L)	15.97 ± 6.43	14.35 ± 6.44
Serum GGT (IU/L)**	20.76 ± 10.03	24.53 ± 14.38
Age (years)#	20.12 ± 2.61	22.03 ± 4.29
BMI (kg/m^2^)	19.86 ± 2.29	19.72 ± 2.48
Energy intake (kJ/day)	10,077 ± 3,347 (median, 9,789)	9,732 ± 4,136 (median, 8,905)
Total protein intake (g/day)	88 ± 41 (median, 78)	79 ± 40 (median, 68)
Total fat intake (g/day)	52 ± 25 (median, 47)	47 ± 28 (median, 39)
Total carbohydrate intake (g/day)	389 ± 112 (median, 366)	381 ± 151 (median, 344)
Zinc intake (mg/day)	8.7 ± 3.6 (median, 8.4)	8.84 ± 5.0 (median, 7.3)
Vitamin A intake (RE/day)	353.4 ± 315.6 (median, 242.0)	460.6 ± 793.7 (median, 202.0)

Abbreviations: ALT, alanine aminotransferase; AST, aspartate aminotransferase; GGT, γ-glutamyltransferase.

a Association between DDT uptake and RBP status (χ^2^ = 27.4).

* *p*< 0.05;

** *p* < 0.005; and

# *p* < 0.001, compared with unexposed controls.

**Table 2:** Changes in thyroid hormone transport related to DDT uptake and RBP status (mean ± SD).

	DDT isomer concentration (μg/g lipid)			
	< 0.02	≥ 0.02		
Thyroid hormone	Normal RBP^a^ (*n* = 40)	Normal RBP (*n* = 127)	Relatively low RBP (*n* = 151)	Trend
TTR (mg/L)	23.74 ± 5.74	26.72 ± 6.61	20.79 ± 4.66	Quadratic#
Serum fT_4_ (pmol/L)	15.55 ± 2.10	13.88 ± 2.33	12.32 ± 2.24	Linear#
Serum fT_3_ (pmol/L)	5.55 ± 0.78	5.08 ± 1.15	4.65 ± 1.19	Linear#
Serum TSH (mIU/L)	2.67 ± 1.32	2.10 ± 1.20	2.09 ± 0.98	Quadratic*

Polynomial contrasts were determined on ranked data to assess significance of linear or quadratic trend.

a Normal in 38 of 40 subjects.

* *p* < 0.05, and

# *p* < 0.001.

**Table 3:** Comparison of DDT isomer concentrations of subjects with normal RBP and those with relatively low RBP within the group with evidence of DDT uptake [median (interquartile range)].

	RBP concentration (μg/g lipid)	
DDT isomer	Normal (*n* = 127)	Relatively low (*n* = 151)
*o*,*p*′-DDT	0.24 (0.00–0.98)	0.45 (0.05–1.36)*
*p*,*p*′-DDT	1.97 (1.04–12.00)	8.87 (3.82–38.50)#
*o*,*p*′-DDD	0.00 (0.00–0.29)	0.05 (0.00–0.28)
*p*,*p*′-DDD	0.00 (0.00–0.57)	0.01 (0.00–0.43)
*o*,*p*′-DDE	0.00 (0.00–0.04)	0.01 (0.00–0.47)#
*p*,*p*′-DDE	0.92 (0.00–54.54)	49.57 (26.13–150.43)#
∑DDT	5.39 (1.36–75.04)	63.51 (33.47–211.50)#
*p*,*p*′-DDE/*p*,*p*′-DDT	0.66 (0.00–3.51)	3.82 (2.08–7.21)#

Comparisons were performed with the Wilcoxon rank-sum test on log-transformed data.

* *p* < 0.05, and

# *p* < 0.001, compared with the normal RBP group.
